# Distribución y caracterización fenotípica y genotípica de *Listeria monocytogenes* en aislamientos de alimentos, Colombia, 2010-2018

**DOI:** 10.7705/biomedica.6152

**Published:** 2021-10-15

**Authors:** Ana Isabel Muñoz, Edna Catering Rodríguez

**Affiliations:** 1 Instituto Nacional de Vigilancia de Medicamentos y Alimentos, INVIMA, Bogotá, D.C., Colombia Instituto Nacional de Vigilancia de Medicamentos y Alimentos, INVIMA Bogotá, D.C. Colombia

**Keywords:** Listeria monocytogenes, enfermedades transmitidas por alimentos, electroforesis en gel de campo pulsado, Listeria monocytogenes, foodborne diseases, electrophoresis, gel, pulsed-field

## Abstract

**Introducción.:**

*Listeria monocytogenes* es un patógeno transmitido por alimentos que causa infecciones en humanos, entre ellas, meningitis, meningoencefalitis y septicemias, así como abortos. Con la tipificación serológica se han identificado 13 serotipos, siendo el 4b el causante de la mayoría de los brotes en el mundo.

**Objetivo.:**

Determinar la frecuencia y la distribución de los serotipos y subtipos moleculares de *L. monocytogenes* aislados de alimentos en Colombia entre el 2010 y el 2018.

**Materiales y métodos.:**

Se hizo un estudio descriptivo y retrospectivo a partir del análisis de 2.420 aislamientos que fueron identificados como *L. monocytogenes* y otras especies, por medio de pruebas bioquímicas, serológicas y de subtipificación molecular mediante electroforesis en gel de campo pulsado (PFGE).

**Resultados.:**

De los 2.420 aislamientos recibidos, 2.326 fueron confirmados como *L. monocytogenes*. Los serotipos encontrados fueron: 4b (52%), 4d-4e (14,5%), 1/2a (11%), 1/2c (9,4%), 1/2b (9 %), y 3a, 3b, 3c, 4c, 4d, 4e y 7 (menos de 2%). Procedían de Bogotá (43%), Antioquia (25%), Valle (10%), Nariño (9%) y otros departamentos (7%). La caracterización genotípica agrupó los aislamientos evaluados en 167 patrones de PFGE; los perfiles más frecuentes se presentaron en productos lácteos, cárnicos y alimentos preparados.

**Conclusión.:**

El 96,1 % de los aislamientos correspondieron a *L. monocytogenes*, con una buena concordancia entre el aislamiento y la identificación; el serotipo 4b, extremadamente virulento, fue el más frecuente. El análisis molecular evidenció la posible diseminación y permanencia en el tiempo de varios serotipos, lo que resalta la importancia de incluir este patógeno en los programas de vigilancia epidemiológica en alimentos.

## Abstract (en)

**Introduction:**

*Listeria monocytogenes* is a food-borne pathogen that may cause infections in humans such as meningitis, meningoencephalitis, and septicemia, as well as abortions. By serological typing 13 serotypes have been identified of which 4b is responsible for most of the outbreaks in the world.

**Objective:**

To determine the frequency and distribution of serotypes and molecular subtypes of L. monocytogenes isolated in Colombia from food from 2010 to 2018.

**Materials and methods:**

We conducted a retrospective and descriptive study based on the analysis of 2,420 isolates confirmed as *L. monocytogenes* and other species using biochemical and serological tests, and pulsed-field gel electrophoresis (PFGE) for molecular subtyping.

**Results:**

Of the 2,420 isolates received, 2,326 were confirmed as *L. monocytogenes*. The serotypes found were 4b (52%), 4d-4e (14.5%), 1/2a (11%), 1/2c (9.4%), 1/2b (9%), and 3a, 3b, 3c, 4c, 4d, 4e and 7 (less than 2%). The isolates came from Bogotá (43%), Antioquia (25%), Valle (10%), Nariño (9%), and other departments (7%). The genotypic characterization grouped the isolates in 167 PFGE patterns. The most frequent patterns were identified in various dairy and meat products, and in prepared foods.

**Conclusion:**

A 96.1% of the isolates corresponded to *L. monocytogenes* showing good agreement between isolates and identification. Serotype 4b, highly virulent, was the most frequent. The molecular analysis showed the possible dissemination and permanence over time of several serotypes, which highlights the importance of including this pathogen in epidemiological food surveillance programs.

El género *Listeria* está conformado por bacterias Gram positivas con un bajo contenido de G+C ^1^. Hasta 1984, se incluían en él seis especies ^1^: *L. monocytogenes*, *L. innocua*, *L. seeligeri*, *L. welshimeri*, *L. grayi* y *L. ivanovii*^2^^-^^6^. En el 2009, se describieron otras dos especies: *L. rocourtiae* y *L. marthii*^7^^-^^8^, y posteriormente, se reconocieron nueve más: *L. aquatica*, *L. cornellensis*, *L. floridensis*, *L. grandensis*, *L. riparia*, *L. booriae*, *L. newyorkensis*, *L. fleischmannii* y *L. weihenstephanensis*^9^^-^^12^. Recientemente, mediante estudios filogenéticos y a partir de la información de la secuencia genómica completa, se identificaron las siguientes nuevas especies: *L. costaricencis*, *L. goaensis*, *L. thailandesis*, *L. valentina*, *L. cossartiae*, *L. farberi*, *L. immoviles*, *L. portnoyi* y *L. rustica*^13^^-^^17^.

*Listeria monocytogenes* se considera una especie patógena para los humanos y los animales, y *L. ivanovii*, para los animales ^1^. No obstante, existen reportes de listeriosis humanas ocasionadas por *L. ivanovi* y *L. innocua*^1^^,^^18^^,^^19^.-*L. monocytogenes* es un microorganismo ubicuo, facultativo, intracelular y oportunista, que causa graves infecciones en humanos y animales ^1^. Su principal ruta de transmisión es el consumo de alimentos contaminados ^20^. Tiene la capacidad de adaptarse a condiciones extremas, lo que permite su supervivencia en la cadena de producción de una gran variedad de alimentos de origen animal y vegetal ^20^^,^^21^. Puede ocasionar grandes pérdidas económicas en la industria a nivel nacional e internacional, dada la obligación de retirar del mercado los alimentos contaminados con el microorganismo ^22^^,^^23^.

Este patógeno causa infecciones sistémicas graves, como meningitis, meningoencefalitis, encefalitis y septicemia ^1^^,^^20^^,^^24^^-^^26^. La diseminación bacteriana subclínica puede causar endocarditis, e infecciones cutáneas, osteoarticulares o de las vías biliares ^24^. Además, las infecciones de la madre y del recién nacido pueden producir aborto espontáneo, meningitis, septicemia neonatal e, incluso, la muerte fetal ^1^^,^^20^^,^^24^^-^^27^.

*Listeria monocytogenes* ocupa el tercer lugar como agente etiológico causante de meningitis neonatal, con una letalidad de hasta el 60% ^27^. Las mujeres embarazadas tienen diez veces más probabilidades de contraer infecciones por listeriosis que otras personas ^25^.

A pesar de que la listeriosis continúa siendo una enfermedad poco frecuente, con una morbilidad baja comparada con otras enfermedades transmitidas por alimentos, la mortalidad por esta causa está entre el 20 y el 30% ^1^^,^^23^^,^^26^^,^^28^, y en el 95% de los casos requiere hospitalización ^28^.

En el mundo se han reportado muchos brotes de listeriosis humana asociados con diferentes tipos de alimentos, como quesos y otros derivados lácteos, frutas (melones), productos cárnicos, mariscos y pescados ahumados ^20^^,^^29^^-^^36^.

Para identificar las cepas de *L. monocytogenes*, se utilizan diferentes métodos fenotípicos como las pruebas bioquímicas de identificación y confirmación de especies ^37^; y la de tipificación serológica, muy útil para determinar los serotipos mediante el esquema de Paterson. Esta metodología, que fue modificada por Seeliger y Hohne ^38^^,^^39^ y aún está vigente, ha permitido caracterizar 13 serotipos de la especie: 1/2a, 1/2b, 1/2c, 3a, 3b, 3c, 4a, 4ab, 4b, 4c, 4d, 4e y 7 ^1^^,^^23^^,^^26^^,^^28^^,^^38^^,^^39^.

Para la caracterización genotípica de *L. monocytogenes* en este estudio, se utilizó la electroforesis en gel de campo pulsado (*Pulsed-Field Gel Electrophoresis*, PFGE). En el estudio multicéntrico de subtipificación de *L. monocytogenes* de la Organización Mundial de la Salud (OMS) de 1996, se determinó que la PFGE era un método preciso y reproducible para comparar molecularmente los aislamientos de este agente patógeno, utilizando las enzimas de restricción *Asc*l y *Apa*l ^40^.

En Colombia, la listeriosis no es una enfermedad de notificación obligatoria; sin embargo, en el periodo 2010-2018 se notificaron al Sistema de Vigilancia en Salud Pública (Sivigila) 42 brotes de enfermedad transmitida por alimentos ocasionados por *L. monocytogenes*, los cuales afectaron a 1.255 personas por el consumo de alimentos como quesos frescos, derivados cárnicos, comidas de restaurantes y, en general, alimentos listos para el consumo ^41^.

Para el estudio, se hizo una búsqueda institucional activa en el Sistema de Información de Apoyo a la Notificación e Investigación de Eventos de Interés en Salud Pública (SIANIESP) del Sivigila en los registros individuales de prestación de servicios de salud (RIPS), para: listeriosis cutánea (A230), otras formas de listeriosis (A328), listeriosis no específica (A329), listeriosis congénita (P372), meningitis y meningoencefalitis (A321), y septicemias (A327). Hubo un total de 696 consultas externas por listeriosis, de las cuales 487 fueron por meningitis y meningoencefalitis, y 209 por las otras formas de listeriosis. Asimismo, en este periodo los servicios de urgencias notificaron al Sivigila 85 casos de listeriosis ^42^.

En Colombia, la meningitis bacteriana es una enfermedad de notificación obligatoria cuando el agente causal es *Haemophillus influenzae, Streptococcus pneumonie* o *Neisseria meningitidis*, pero no cuando es *L. monocytogenes*, lo cual ocasiona un vacío en la vigilancia de este microorganismo ^43^. El Instituto Nacional de Vigilancia de Medicamentos y Alimentos (Invima), consciente de la gravedad de la listeriosis y teniendo en cuenta que aún no está contemplada en la vigilancia de los alimentos, ha incluido *L. monocytogenes* como un indicador obligatorio de inocuidad en la vigilancia de los alimentos que se producen o se consumen en el país. Esta vigilancia es rutinaria y es ejercida por las entidades territoriales; además, el envío de los aislamientos de los laboratorios de salud pública al laboratorio de referencia del Invima para su confirmación, serotipificación y subtipificación molecular, es de obligatorio cumplimiento.

En este contexto, los objetivos de este estudio fueron confirmar el resultado de los aislamientos de *L. monocytogenes* enviados por los laboratorios de salud pública, y determinar la frecuencia y distribución de los serotipos y subtipos moleculares que circulan en el país procedentes de los alimentos que son motivo de vigilancia, como también, los de aquellos asociados epidemiológicamente con los brotes en el periodo comprendido entre el 2010 y el 2018.

## Materiales y métodos

### Tipo de estudio

Se hizo un estudio descriptivo y retrospectivo entre el 2010 y el 2018 para confirmar la presencia de *L. monocytogenes* en los aislamientos recibidos en el Laboratorio de Microbiología de Alimentos del Invima.

### Muestra

Durante este periodo, se recibieron 2.420 aislamientos de *L. monocytogenes* enviados por los laboratorios de alimentos de los departamentos adscritos a los de salud pública (LDSP) de Arauca, Antioquia, Atlántico, Bolívar, Boyacá, Caldas, Caquetá, Casanare, Cauca, Cesar, Córdoba, Cundinamarca, Bogotá, La Guajira, Magdalena, Meta, Nariño, Norte de Santander, Putumayo, Quindío, Risaralda, San Andrés Islas, Santander, Sucre y Valle del Cauca, además de los aislamientos recuperados en el laboratorio de referencia. Los servicios de Amazonas, Guainía, Guaviare, Huila, Tolima, Vaupés y Vichada, no enviaron ningún aislamiento.

En el análisis de las variables, se utilizó el programa Microsoft Office Excel 2013^®^ para calcular las frecuencias y las proporciones, y luego se hicieron un análisis univariado y uno bivariado de los datos del estudio. Se incluyeron las siguientes variables: categoría de alimentos, alimentos, procedencia, serotipos y patrones de PFGE.

### Caracterización fenotípica

Para identificar los aislamientos se siguió el procedimiento del manual de bacteriología analítica (*Bacteriologycal Analytical Manual*): coloración de Gram, determinación de catalasa, pruebas bioquímicas (ramnosa, xilosa y manitol), crecimiento en forma de sombrilla en agar, motilidad, pruebas de hemólisis y prueba de Christie-Atkins-Much-Peterson (CAMP). Asimismo, se utilizó el sistema bioquímico API *Listeria de Biomérieux*^37^.

Para la tipificación serológica de *Listeria* spp., se siguió el protocolo de Seeliger y Hohne ^38^ desarrollado por el Laboratorio de *Listeria* del Instituto Pasteur en París, Francia ^44^. Se incluyeron antisueros de factores somáticos y flagelares producidos por el Laboratorio de Microbiología de Alimentos del Invima, y se utilizaron cepas de referencia del Instituto Pasteur de París mediante la inmunización de conejos Nueva Zelanda; asimismo, se utilizaron los antisueros comerciales Eurobio™ y Denka Seiken™.

El control externo de calidad de la identificación y serotipificación, y su seguimiento, estuvieron a cargo del Laboratorio de *Listeria* y del Centro Colaborativo de la OMS para la listeriosis de origen alimentario del Instituto Pasteur de París.

Los alimentos de los cuales se aisló *L. monocytogenes* se clasificaron según su categoría y procedencia.

### Caracterización genotípica

Los aislamientos de *L. monocytogenes* se caracterizaron con la técnica de PFGE ya descrita ^45^. Se seleccionó la enzima de restricción *Apa*l, teniendo en cuenta que el poder discriminatorio calculado mediante el índice de Simpson no difiere entre las dos enzimas recomendadas, *Apa*l y *Asc*l, cuando se evalúa *L. monocytogenes*^46^; además, se ha demostrado que el número de perfiles únicos observado con *Apa*l es mayor que el obtenido con *Asc*l ^47^, y se ha evidenciado que el análisis del serotipo 4b con la enzima *Apa*l tiene mayor poder de discriminación que otros métodos de tipificación, incluida la restricción con *Asc*l ^48^. Los dendrogramas se generaron con el programa Bionumérics™, versión 7.5 (Applied Maths, Bélgica) mediante el método UPGMA con una optimización de tolerancia de 1,5%.

La muestra para seleccionar los aislamientos sometidos a PFGE, se calculó utilizando Epi-Info, versión 7.2, de 2018, con una estimación del 50% (por ser desconocida), un error esperado del 5 % y una significación del 95%. Se le atribuyó un peso porcentual a cada categoría de alimento y a la frecuencia de sus serotipos. La selección fue aleatoria y quedó conformada por 368 aislamientos distribuidos así: leche y derivados lácteos (n=188), carne y derivados cárnicos (n=93), alimentos listos para el consumo (n=30), aves (n=15), muestras de plantas procesadoras de alimentos (n=3), y productos de panadería y pastelería (n=1). Además, se analizaron todos los aislamientos (n=38) implicados en casos y brotes de enfermedades transmitidas por alimentos.

## Resultados

De los 2.420 aislamientos recibidos, *L. monocytogenes* se confirmó en 2.326 (96,1%) y se identificaron otras especies del género *Listeria* en 94 (3,9%). De los 2.326 de *L. monocytogenes*, 2.288 (98,4%) provenían de alimentos que son objeto de los programas de vigilancia del Invima como parte del control de calidad de alimentos en el país, y los 38 (1,6%) restantes correspondían a casos y brotes de enfermedades transmitidas por alimentos.

**Figura 1:**
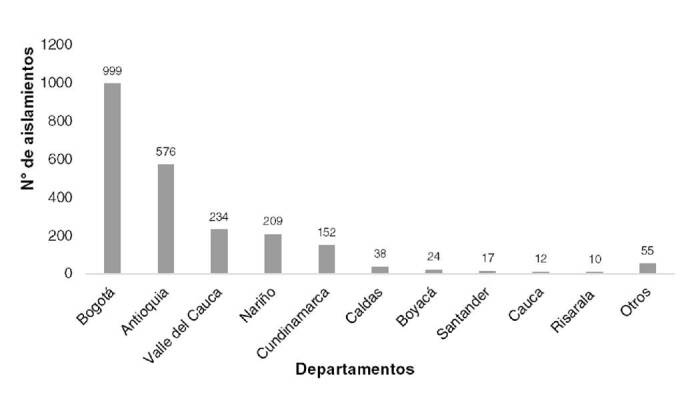
Presencia de *Listeria monocytogenes* aislada de alimentos según su procedencia, Colombia 2010-2018

Los alimentos remitidos y confirmados como *L. monocytogenes* por el laboratorio del Invima procedían, con mayor frecuencia, de Bogotá, Antioquia y Valle del Cauca (figura 1).

Las categorías de alimentos más frecuentemente contaminados por *L. monocytogenes* fueron las leches y los derivados lácteos (n=1.317; 56,6%), las carnes y los derivados cárnicos (n=659; 28,3%) y los alimentos listos para el consumo (n=209; 9%) (cuadro suplementario 1).

Las categorías de alimentos en los que más frecuentemente se aisló *L. monocytogenes* fueron las leches y los derivados lácteos, con 19,91 % en Antioquia y 17,41% en Bogotá, y las carnes y derivados cárnicos, con 17,37% en Bogotá (cuadro suplementario 2).

**Figura 2:**
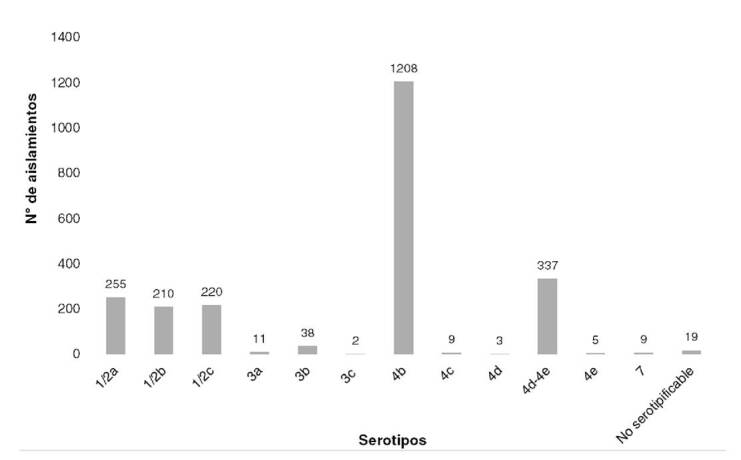
Determinación de serotipos de *Listeria monocytogenes* aislados de alimentos, Colombia, 2010-2018

Durante el periodo de estudio, se determinaron 12 serotipos. El serotipo más frecuente fue el 4b (n=1.208) (figura 2), y la mayor incidencia de este serotipo se encontró, en primer lugar, en la categoría de leches y derivados lácteos (n=711) y, en segundo lugar, en la de carnes y derivados cárnicos (n=327) (cuadro suplementario 1).

Los quesos campesinos fueron el alimento con mayor frecuencia (31,34%) de aislamientos de *L. monocytogenes*. El serotipo aislado más veces en el grupo completo de alimentos, fue el 4b (51,93%) (cuadro 1).

**Cuadro 1:** Frecuencia de *Listeria monocytogenes* y determinación de serotipos aislados de alimentos, Colombia, 20102018

Alimentos	1/2a	1/2b	1/2c	3a	3b	3c	4b	4c	4d	4d-4e	4e	7	NS	Total
	%	%	%	%	%	%	%	%	%	%	%	%	%	%
Queso campesino	2,24	2,19	4,56	0,39	0,77	0,04	16,17	0,00	0,04	4,69	0,00	0,04	0,21	31,34
Jamón cocido de cerdo, cordero, pavo y pollo	1,03	1,76	0,95	0,00	0,13	0,00	6,15	0,09	0,04	1,29	0,04	0,04	0,09	11,61
Cuajada	0,73	0,26	1,12	0,00	0,04	0,00	2,49	0,00	0,00	0,60	0,00	0,00	0,04	5,29
Leches crudas	0,52	0,09	0,04	0,00	0,04	0,04	2,71	0,00	0,00	0,52	0,00	0,00	0,00	3,96
Salchichas cocidas	0,39	0,34	0,21	0,00	0,17	0,00	1,16	0,00	0,04	0,52	0,00	0,00	0,00	2,84
Jamón de cerdo ahumado o madurado	0,90	0,04	0,17	0,00	0,00	0,00	1,16	0,00	0,00	0,39	0,00	0,13	0,04	2,84
Queso mozzarella	0,21	0,26	0,00	0,00	0,00	0,00	1,72	0,04	0,00	0,30	0,00	0,04	0,09	2,67
Sánduche de pollo, jamón, y queso	0,09	0,13	0,30	0,00	0,00	0,00	1,20	0,04	0,00	0,69	0,00	0,00	0,00	2,45
Mortadela	0,26	0,09	0,04	0,00	0,17	0,00	1,42	0,00	0,00	0,39	0,00	0,04	0,00	2,41
Ensalada de verduras	0,26	0,17	0,00	0,00	0,00	0,00	1,07	0,00	0,00	0,34	0,00	0,00	0,00	1,85
Total	6,62	5,33	7,39	0,39	1,33	0,09	35,25	0,17	0,13	9,72	0,04	0,30	0,47	67,24
Otros	4,34	3,70	2,06	0,09	0,30	0,00	16,68	0,21	0,00	4,77	0,17	0,09	0,34	32,76
Total	10,96	9,03	9,46	0,47	1,63	0,09	51,93	0,39	0,13	14,50	0,21	0,39	0,82	100,00

NS: no serotipificable

De los 368 aislamientos seleccionados para PFGE, la técnica pudo utilizarse en 333; no fue posible hacerlo en 35 (9,5%), probablemente por la calidad del ADN. Estos aislamientos se agruparon en 167 patrones de PFGE diferentes y se designaron de LM-001 a LM-167 (figura suplementaria 1).

### Distribución de patrones frecuentes de PFGE

Para el análisis de los patrones de PFGE obtenidos, se hizo una clasificación de los cuatro patrones más frecuentes, es decir, aquellos con más de 10 aislamientos (cuadro 2) (figura suplementaria 1).

*Patrón LM-076*. El 81,2% (13/16) de los aislamientos pertenecía al serotipo 4b, el cual se presentó en siete clases diferentes de alimentos y en tres departamentos (cuadro 2) (figura suplementaria 1). Este patrón únicamente se presentó en los cinco primeros años de vigilancia.

*Patrón LM-0154*. El 87,5% (14/16) de los aislamientos con este patrón correspondió al serotipo 1/2c, el cual fue el más representativo, seguido por el 1/2b y el 4b. El queso campesino fue el alimento en el cual se encontró con mayor frecuencia y las muestras provenían casi exclusivamente del departamento de Antioquia (14/16) (87,5%) (cuadro 2) (figura suplementaria 1). Este patrón únicamente se presentó en los cinco primeros años de vigilancia (cuadro 3).

**Cuadro 2:** Distribución de perfiles de PFGE más frecuentes según tipo de alimento, Colombia, 2010-2018

Patrón PFGE	Alimento relacionado	Número de aislamientos por patrón	Serotipos								Procedencia
			1/2a	1/2b	1/2c	3a	3b	4b	4d	NS
LM 076	Ensalada de verduras (1), helado (1), jamón (3), leche cruda (1), pollo crudo (1), queso campesino (8) y queso doble crema (1)	16	1	0	0	0	0	13	0	2	Antioquia (9) Bogotá (5) Caldas (2)
LM 154	Cuajada (1), jamón de cerdo (1), queso campesino (14)	16	0	1	14	0	0	1	00	0	Antioquia (14) Bogotá (2)
LM 047	Agua de panela (1), carne cocinada (1), carne cruda (1), crema de leche (1), flan de leche (1), jamón (2), queso campesino (2), salami (1), sánduche (1), tocineta (1)	12	0	0	0	0	0	6	6	0	Bogotá (11) Valle (1)
LM 072	Arroz con pollo (1), ensalada de frutas (1), ensalada de verduras (1), jamón cocido (1), menú (1), pechuga de pollo cocinada (1), queso campesino (4)	10	0	0	0	0	0	10	0	0	Antioquia (6) Bogotá (1) Caldas (1) Cundinamarca (1) Valle (1)
Otros patrones n=163	67 diferentes tipos de alimentos, el queso campesino concentró el mayor número de muestras: 69 en total	279	34	37	21	2	5	146	32	2	Antioquia (66) Bogotá (108) Nariño (36) Valle (28) Cundinamarca (9) Caldas (7) San Andrés (3) Amazonas, Boyacá, Cauca, Magdalena, Quindío, Risaralda y Putumayo (2 aislamientos cada uno) Arauca, Bolívar, Caquetá, Cesar, Córdoba, Magdalena, Norte de Santander, San Andrés (1 aislamiento cada uno)
Total de aislamientos		333	35	38	35	2	5	176	38	4	

NS: no serotipificable

**Cuadro 3:** Distribución de perfiles de PFGE más frecuentes por serotipo y año en aislamientos de *Listeria monocytogenes*, Colombia, 2010-2018

Año/patron de PFGE	Serotipos																	Número de aislamientos
	1/2a		1/2b		1/2c		3a	3b	4b					4d		NS		
	76	O	154	O	154	O	O	O	47	72	76	154	O	47	O	76	O	
2010	0	5	1	11	2	1	1	2	0	0	1	1	32	3	7	0	0	67
2011	0	8	0	9	2	3	1	1	1	3	1	0	25	2	6	0	0	62
2012	0	5	0	3	4	5	0	2	1	0	5	0	20	0	2	1	1	49
2013	0	7	0	5	2	3	0	0	0	5	6	0	18	0	8	0	0	54
2014	1	4	0	6	4	6	0	0	0	1	0	0	23	1	8	1	1	56
2015	0	2	0	1	0	1	0	0	1	1	0	0	11	0	1	0	0	18
2016	0	0	0	1	0	1	0	0	0	0	0	0	7	0	0	0	0	9
2017	0	2	0	0	0	1	0	0	2	0	0	0	7	0	0	0	0	12
2018	0	1	0	1	0	0	0	0	1	0	0	0	3	0	0	0	0	6
Total	1	34	1	37	14	21	2	5	6	10	13	1	146	6	32	2	2	333

NS: no serotipificable; patrón de PFGE: (047/072/076/154); O: otros, corresponde a 163 perfiles que contenían menos de 10 aislamientos cada uno

*Patrón LM-047*. Se encontraron 12 aislamientos con este patrón, seis del serotipo 4b y seis del 4d, en 10 alimentos diferentes y casi exclusivamente (91,6%) en Bogotá (cuadro 2) (figura suplementaria 1).

*Patrón LM-072*. Todos los aislamientos de este patrón fueron del serotipo 4b, es decir que, en las muestras evaluadas en el estudio, este patrón se detectó exclusivamente en este serotipo. Se presentó en siete clases de alimentos diferentes y en cinco departamentos, en los años 2011, 2013, 2014 y 2015 (cuadro 2) (figura suplementaria 1).

*Otros patrones*. Se encontraron 163 patrones diferentes de PFGE en 279 aislamientos distribuidos en 67 diferentes tipos de alimentos, de los cuales el más frecuente fue el queso campesino. Los serotipos más frecuentes en esta categoría fueron el 4b, con 146 (52,4%) aislamientos, el 1/2b, con 37 (13,2%), el 1/2a, con 34 (12,2%), y el 4d, con 32 (11,5%). Los aislamientos de esta categoría procedían de 22 departamentos (cuadro 2) (figura suplementaria 1).

### Distribución de patrones de PFGE por serotipo

*Serotipo 4b*. De los 333 aislamientos evaluados por PFGE, más de la mitad (n=176; 52,9%) correspondió al serotipo 4b, recuperados durante todos los años de vigilancia (cuadro 3).

*Serotipo 4d*. Se procesaron 38 (11,4%) aislamientos recuperados en el 2010, el 2011 y el 2014 (cuadro 3).

*Serotipo 1/2b*. Se procesaron 38 (11,4%) aislamientos recuperados en todos los años, excepto en el 2017. Solo uno se relacionó con los patrones frecuentes.

*Serotipos 1/2a y 1/2c*. Del serotipo 1/2a se analizaron 35 (10,5%) aislamientos encontrados en todos los años de estudio, excepto en el 2016, en tanto que el serotipo 1/2c se evaluó en todos los años de estudio, excepto en el 2018 (cuadro 3).

*Otros serotipos*. Los 11 aislamientos restantes correspondieron a los serotipos 3b (1,5%) y 3a (0,6%), y cuatro (1,2%) no pudieron ser serotipificados (cuadro 3). Del serotipo 4c no se evaluaron aislamientos con la PFGE.

### Patrones de PFGE en aislamientos causantes de enfermedades transmitidas por alimentos

Los 38 aislamientos clasificados en esta categoría fueron relacionados epidemiológicamente con enfermedades transmitidas por alimentos por los laboratorios de salud pública. Se evaluaron 35 aislamientos mediante PFGE, los cuales se distribuyeron en cuatro serotipos y 30 patrones.

*Serotipo 1/2a*. Se encontraron cuatro aislamientos distribuidos en cuatro patrones de PFGE.

*Serotipo 1/2b*. Se encontraron cuatro aislamientos distribuidos en cuatro patrones de PFGE.

*Serotipo 1/2c*. Se encontraron siete aislamientos distribuidos en cinco patrones de PFGE.

**Figura 3:**
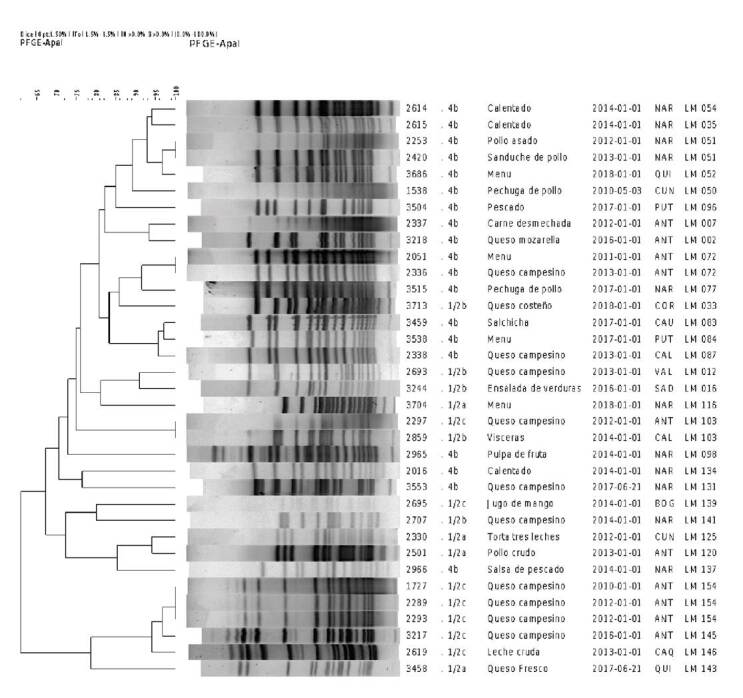
Patrones de PFGE de aislamientos de *Listeria monocytogenes* relacionados con brotes de enfermedades transmitidas por alimentos, Colombia, 2010-2018

*Serotipo 4b*. Se encontraron 20 aislamientos distribuidos en 18 patrones de PFGE (figura 3). Los serotipos 3a, 3b, 4c y 4d no se relacionaron enfermedades transmitidas por alimentos.

Los brotes de enfermedades transmitidas por alimentos se relacionaron con las categorías de alimentos preparados (arroz con pollo, calentado, carne desmechada, jugo, tortas), derivados lácteos (leche cruda, queso) y derivados cárnicos (pollo) (figura 3). Los departamentos que enviaron mayor cantidad de aislamientos relacionados con este tipo de enfermedades durante este periodo de estudio, fueron Antioquia y Nariño.

Dos aislamientos del serotipo 4b presentaron el patrón LM-51 y ambos provenían del departamento de Nariño; se encontraron en alimentos que contenían pollo y se enviaron en dos años diferentes, lo cual demuestra la persistencia en el tiempo de este patrón y su relación con enfermedades transmitidas por alimentos.

Una situación similar se presentó en Antioquia con el patrón 072, el cual se recuperó en el 2011 y en el 2013 en alimentos relacionados con estas enfermedades; el patrón LM-154 se encontró en tres aislamientos con el serotipo 1/2c, todos procedentes de queso campesino en dos años diferentes.

## Discusión

En Colombia, la autoridad sanitaria ha incluido *L. monocytogenes* en la vigilancia rutinaria de los alimentos como indicador de inocuidad, por ser un microorganismo causante de enfermedades transmitidas por alimentos, aunque aún no se encuentra regulado en las normas nacionales.

Los serotipos encontrados en este estudio, el 4b, el 1/2a y el 1/2b, coinciden con los reportados a nivel mundial relacionados con casos o brotes de listeriosis ^1^^,^^21^^,^^23^^,^^24^^,^^26^^,^^28^. Según el ciclo evolutivo de *L. monocytogenes*, hay cuatro linajes. Los serotipos 4b y 1/2b pertenecen al linaje I y son causantes de la mayoría de las listeriosis humanas transmitidas por alimentos; el serotipo1/2a pertenece al linaje II, implicado en brotes de listeriosis humanas y también aislado del ambiente y de alimentos ^49^. Aunque no todos los serotipos de *L. monocytogenes* tienen factores de virulencia similares, para efectos de la salud pública, todos ellos se consideran potencialmente patógenos ^1^^,^^23^^,^^26^.

En la vigilancia rutinaria de alimentos, Bogotá fue la entidad territorial que envió el mayor número de aislamientos de *L. monocytogenes*, seguida por Antioquia, lo que posiblemente se debe a que existe un mejor programa de vigilancia de este agente patógeno en estas dos seccionales.

Los derivados lácteos, principalmente el queso campesino y las cuajadas, fue el grupo de alimentos en el que más se aisló *L. monocytogenes*, principalmente del serotipo 4b, lo cual también puede deberse a que muchos son de producción artesanal, en la cual es más común el uso de leches no pasteurizadas, así como la ausencia de buenas prácticas de manufactura.

En los derivados cárnicos, las concentraciones de nitritos permitidas como conservantes no inhiben a *L. monocytogenes*^31^ y permiten su crecimiento. En el presente estudio se reportaron aislamientos, principalmente del serotipo 4b, en jamones cocidos de cerdo, cordero, pavo y pollo. Es probable que esto se deba a fallas en el mantenimiento de la higiene a lo largo de la cadena de producción, conservación, transporte, fraccionamiento y manipulación en la venta de estos productos, lo cual, unido a características como el gran contenido de nutrientes, la importante actividad del agua (AW) (≥0,92%), un pH de 4,4 o menos, y la conservación prolongada a bajas temperaturas de refrigeración, permite la supervivencia de *L. monocytogenes*^50^.

En comparación con un reporte previo de la distribución de serotipos de *L. monocytogenes* en alimentos en Colombia ^51^, se encontró que no se han presentado variaciones en el tipo de alimento contaminado con este agente patógeno con respecto a los años anteriores y, tampoco, en los serotipos encontrados, lo cual pone en evidencia la falta de intervención en los establecimientos que son de obligatoria vigilancia y control.

Se ha sugerido que en los aislamientos de alimentos se encuentran principalmente los serotipos 1/2a,1/2b y 1/2c ^1^^,^^21^. En el presente estudio, se observó con mayor frecuencia el serotipo 4b, de gran virulencia y causante de la enfermedad en humanos ^1^^,^^21^^,^^23^^,^^26^, como ya lo reportaron Montero, *et al*. ^52^, quienes evaluaron los serotipos de *L. monocytogenes* aislados de alimentos listos para el consumo y encontraron una asociación del serotipo 4b con alimentos como el queso y los mariscos congelados. Esto puede deberse a que pertenecen a uno de los clones epidémicos identificados que circulan en el mundo, característica que debería evaluarse en estudios posteriores; además, cobra importancia porque uno de los patrones frecuentes encontrados en este estudio, el LM 072, se asoció exclusivamente con aislamientos de serotipo 4b provenientes de alimentos listos para el consumo, como arroz con pollo, ensalada de frutas y verduras, menú listo para consumir, pechuga de pollo cocinada y queso campesino. Ello evidencia la necesidad de incluir a la ausencia de *L. monocytogenes* como parámetro de inocuidad obligatorio, principalmente en la categoría de alimentos listos para el consumo.

Se ha reportado que los aislamientos de origen alimentario tienen mayor diversidad genética que los de origen humano ^53^. Esta característica también se observó en el presente estudio, en el cual se obtuvieron más de 160 perfiles diferentes de electroforesis, lo que sugiere diversas fuentes y condiciones de selección para los genotipos específicos encontrados en alimentos y ambientes de plantas de producción; además, sugiere que solo ciertos grupos de alimentos están relacionados con la enfermedad en humanos ^54^^,^^55^.

Los aislamientos caracterizados mediante PFGE permitieron identificar patrones moleculares que pueden indicar contaminación y persistencia de clones en el tiempo. La persistencia de ciertos genotipos en las instalaciones destinadas a la producción de alimentos, puede ser un signo de fallas en el control del proceso de fabricación o indicar resistencia de los microorganismos a los métodos de limpieza utilizados. Tal persistencia puede deberse a la supervivencia y crecimiento de ciertas cepas en biopelículas o nichos dentro del entorno alimentario, las cuales pueden ser difíciles de limpiar y desinfectar, así como a la reintroducción repetida de tales cepas del ambiente externo en las instalaciones de procesamiento de alimentos a lo largo del tiempo ^54^.

La cantidad de patrones caracterizados como no persistentes, que en este trabajo se clasificaron como “otros”, indica la diversidad de aislamientos que existen en el entorno del procesamiento de alimentos, así como una probable contaminación esporádica continua, lo que indicaría falta de cumplimiento de los protocolos de higiene y limpieza y, por lo tanto, el riesgo de contaminación de los productos alimenticios ^54^.

Los patrones de PFGE clasificados como más frecuentes en este estudio, se presentaron por lo menos una vez en la mayoría de los años en varios departamentos y en alimentos listos para consumir, como quesos, carnes, comidas preparadas y sánduches, entre otros. La permanencia de estos patrones de PFGE en el tiempo, sugiere que los aislamientos de *L. monocytogenes* implicados tienen un reservorio permanente en los intervalos de tiempo, por lo general largos, entre sus apariciones ^53^^,^^54^.

Este estudio brinda información importante sobre la epidemiología de *L. monocytogenes* y puede ayudar a adoptar decisiones sanitarias en Colombia, así como servir de base para estudios posteriores de identificación de los clones epidémicos. Consideramos que los resultados de la genotipificación se pueden utilizar como información de base para hacer análisis genéticos ampliados, por ejemplo, la secuenciación completa del genoma de *L. monocytogenes*.

La clasificación de algunos alimentos, como los 'menús', se dificultó porque la información enviada al laboratorio no estaba completa. Los resultados obtenidos indican la necesidad de implementar buenas prácticas de manufactura con procedimientos estandarizados, en las fábricas artesanales del país ^20^; además, que las autoridades sanitarias del país deben continuar implementando programas eficaces de vigilancia frente a la prohibición de la venta informal de leche cruda, y de la de quesos o cuajadas elaboradas con leche sin pasteurizar.

Se recomienda, al Ministerio de Salud y Protección Social, incluir la listeriosis entre las enfermedades de notificación obligatoria. Asimismo, incluir en la legislación colombiana la ausencia de *L. monocytogenes* como parámetro de inocuidad en el análisis de los alimentos que, por sus características intrínsecas y extrínsecas, permiten su crecimiento, como es el caso de aquellos listos para el consumo.

## Figures and Tables

**Figura suplementaria 1.**
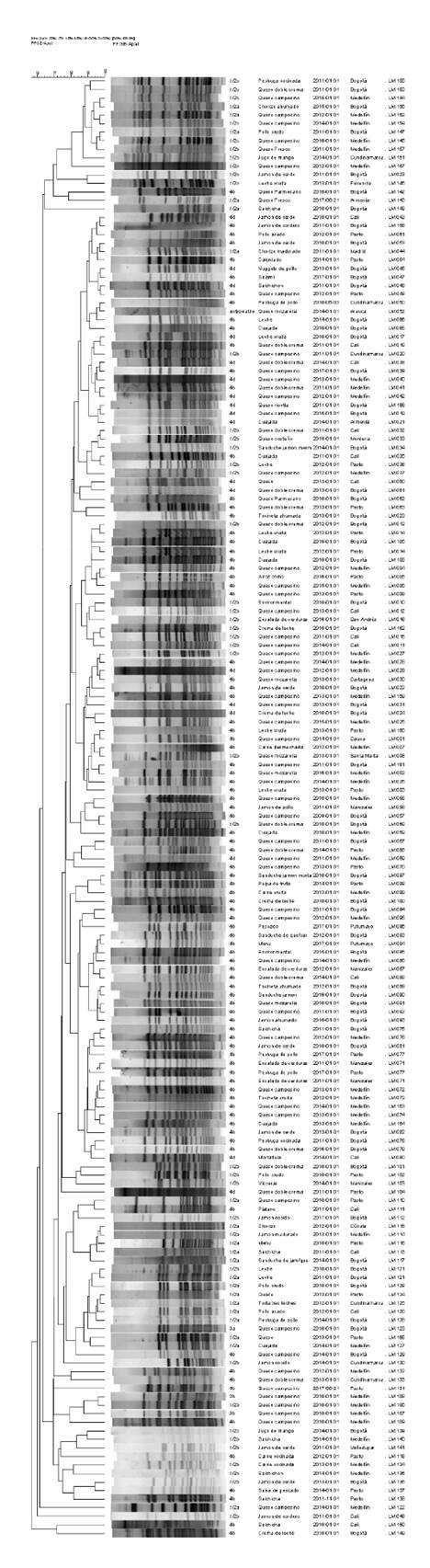
Dendrograma de los 167 patrones únicos de PFGE en aislamientos de *Listeria monocytogenes*, Colombia, 2010-2018

## Archivos suplementarios

**Cuadro suplementario 1.** Frecuencia de *Listeria monocytogenes* y determinación de serotipos aislados de categorías de alimentos, Colombia, 2010-2018

Categorías	1/2a	1/2b	1/2c	3a	3b	3c	4b	4c	4d	4d-4e	4e	7	NS	Total
	N°	N°	N°	N°	N°	N°	N°	N°	N°	N°	N°	N°	N°	N°
Alimentos listos para consumo	25	15	14	0	0	0	115	2	0	35	0	0	3	209
Aves	25	17	10	1	3	0	35	0	0	1	0	0	2	94
Carnes y derivados cárnicos	92	74	50	0	13	0	327	2	2	86	1	5	3	659
Grasas y aceites	0	4	0	0	2	0	2	0	0	0	0	0	0	8
Harinas	0	0	0	0	0	0	1	0	0	0	0	0	0	1
Leches y derivados lácteos	110	90	141	10	19	2	711	5	1	211	4	2	11	1.317
Muestras de plantas procesadoras de alimentos	1	10	1	0	1	0	10	0	0	0	0	2	0	25
Productos no lácteos a base de oleaginosas	0	0	0	0	0	0	0	0	0	1	0	0	0	1
Productos de panadería y pastelería	1	0	0	0	0	0	6	0	0	0	0	3	0	10
Productos derivados de la pesca	1	0	0	0	0	0	1	0	0	0	0	0	0	2
Total	255	210	220	11	38	2	1.208	9	3	334	5	12	19	2.326

NS: no serotipificable

**Cuadro suplementario:** 2. Frecuencia de *Listeria monocytogenes* y determinación de serotipos aislados de categorías de alimentos según su procedencia, Colombia, 2010-2018

Categorías de alimentos	1/2a	1/2b	1/2c	3a	3b	3c	4b	4c	4d	4d-4e	4e	7	NS	Total
	%	%	%	%	%	%	%	%	%	%	%	%	%	%
Bogotá D.C.														
Alimentos listos para el consumo	0,26	0,26	0,52	0,00	0,00	0,00	1,59	0,04	0,00	0,99	0,00	0,00	0,13	3,78
Aves	0,64	0,56	0,43	0,04	0,13	0,00	1,03	0,00	0,00	0,04	0,00	0,00	0,00	2,88
Carnes y derivados cárnicos	2,84	1,42	1,59	0,00	0,17	0,00	8,56	0,09	0,09	2,28	0,04	0,17	0,13	17,37
Leches y derivados lácteos	1,42	1,20	0,95	0,13	0,26	0,00	9,72	0,00	0,04	3,48	0,04	0,09	0,09	17,41
Muestras ambientales de plantas procesadores de alimentos	0,04	0,43	0,00	0,00	0,04	0,00	0,17	0,00	0,00	0,00	0,00	0,09	0,00	0,77
Otras categorías	0,04	0,17	0,00	0,00	0,09	0,00	0,30	0,00	0,00	0,09	0,00	0,00	0,00	0,69
Total	5,25	4,04	3,48	0,17	0,69	0,00	21,37	0,13	0,13	6,88	0,09	0,34	0,34	42,91
**Antioquia**														
Alimentos listos para el consumo	0,00	0,04	0,00	0,00	0,00	0,00	0,43	0,00	0,00	0,00	0,00	0,00	0,00	0,47
Aves	0,13	0,00	0,00	0,00	0,00	0,00	0,04	0,00	0,00	0,00	0,00	0,00	0,00	0,17
Carnes y derivados cárnicos	0,47	0,52	0,60	0,00	0,04	0,00	2,45	0,00	0,00	0,13	0,00	0,00	0,00	4,21
Leches y derivados lácteos	1,20	1,20	4,77	0,09	0,34	0,04	10,06	0,00	0,00	1,98	0,13	0,00	0,09	19,91
Total	1,81	1,76	5,37	0,09	0,39	0,04	12,98	0,00	0,00	2,11	0,13	0,00	0,09	24,76
**Valle del Cauca**														
Alimentos listos para el consumo	0,60	0,09	0,08	0,00	0,00	0,00	0,73	0,04	0,00	0,26	0,00	0,00	0,00	1,76
Aves	0,17	0,00	0,00	0,00	0,00	0,00	0,00	0,00	0,00	0,00	0,00	0,00	0,09	0,26
Carnes y derivados cárnicos	0,21	0,06	0,00	0,00	0,21	0,00	0,82	0,00	0,00	0,99	0,00	0,00	0,00	2,84
Leches y derivados lácteos	0,77	0,64	0,08	0,04	0,09	0,00	2,15	0,09	0,00	1,25	0,00	0,00	0,09	5,16
Productos de panadería y pastelería	0,00	0,00	0,00	0,00	0,00	0,00	0,04	0,00	0,00	0,00	0,00	0,00	0,00	0,04
Total	1,76	1,33	0,15	0,04	0,03	0,00	3,74	0,13	0,00	2,49	0,00	0,00	0,17	10,06
**Nariño**														
Alimentos listos para el consumo	0,09	0,0	0,0	0,00	0,00	0,00	0,73	0,00	0,00	0,09	0,00	0,00	0,00	0,90
Aves	0,04	0,04	0,0	0,00	0,00	0,00	0,21	0,00	0,00	0,00	0,00	0,00	0,00	0,30
Carnes y derivados cárnicos	0,09	0,21	0,04	0,00	0,00	0,00	0,47	0,00	0,00	0,17	0,00	0,00	0,00	0,99
Leches y derivados lácteos	0,69	0,39	0,04	0,04	0,09	0,04	4,08	0,00	0,00	0,99	0,00	0,00	0,13	6,49
Otros	0,00	0,00	0,04	0,00	0,00	0,00	0,26	0,00	0,00	0,00	0,00	0,00	0,00	0,30
Total	0,90	0,64	0,13	0,04	0,09	0,04	5,76	0,00	0,00	1,25	0,00	0,00	0,13	8,99
**Cundinamarca**														
Alimentos listos para el consumo	0,04	0,13	0,04	0,00	0,00	0,00	0,34	0,00	0,00	0,09	0,00	0,00	0,00	0,64
Aves	0,04	0,04	0,00	0,00	0,00	0,00	0,09	0,00	0,00	0,04	0,00	0,00	0,00	0,21
Carnes y derivados cárnicos	0,17	0,09	0,04	0,00	0,00	0,00	0,69	0,00	0,00	0,00	0,00	0,00	0,00	0,99
Leches y derivados lácteos	0,43	0,17	0,09	0,13	0,04	0,00	2,67	0,00	0,00	0,99	0,00	0,00	0,00	4,51
Productos de panadería y pastelería	0,04	0,00	0,00	0,00	0,00	0,00	0,00	0,00	0,00	0,04	0,00	0,00	0,00	0,09
Otros	0,00	0,00	0,00	0,00	0,00	0,00	0,09	0,00	0,00	0,00	0,00	0,00	0,00	0,09
Total	0,73	0,43	0,17	0,13	0,04	0,00	3,87	0,00	0,00	1,16	0,00	0,00	0,00	6,53
Otros														
Total	0,52	0,82	0,21	0,00	0,13	0,00	4,17	0,13	0,00	0,64	0,00	0,04	0,09	6,75
	10,95	9,03	9,46	0,47	1,63	0,09	51,89	0,39	0,13	14,5	0,21	0,39	0,82	100

NS: no serotipificable Nota: los datos presentados corresponden a frecuencias acumuladas.
